# SOS1, HKT1;5, and NHX1 Synergistically Modulate Na^+^ Homeostasis in the Halophytic Grass *Puccinellia tenuiflora*

**DOI:** 10.3389/fpls.2017.00576

**Published:** 2017-04-13

**Authors:** Wei-Dan Zhang, Pei Wang, Zhulatai Bao, Qing Ma, Li-Jie Duan, Ai-Ke Bao, Jin-Lin Zhang, Suo-Min Wang

**Affiliations:** State Key Laboratory of Grassland Agro-Ecosystems, College of Pastoral Agriculture Science and Technology, Lanzhou UniversityLanzhou, China

**Keywords:** *Puccinellia tenuiflora*, *PtSOS1*, *PtHKT1;5*, *PtNHX1*, Na^+^ homeostasis, salt tolerance

## Abstract

*Puccinellia tenuiflora* is a typical salt-excluding halophytic grass with excellent salt tolerance. Plasma membrane Na^+^/H^+^ transporter SOS1, HKT-type protein and tonoplast Na^+^/H^+^ antiporter NHX1 are key Na^+^ transporters involved in plant salt tolerance. Based on our previous research, we had proposed a function model for these transporters in Na^+^ homeostasis according to the expression of *PtSOS1* and Na^+^, K^+^ levels in *P. tenuiflora* responding to salt stress. Here, we analyzed the expression patterns of *PtSOS1*, *PtHKT1;5*, and *PtNHX1* in *P. tenuiflora* under 25 and 150 mM NaCl to further validate this model by combining previous physiological characteristics. Results showed that the expressions of *PtSOS1* and *PtHKT1;5* in roots were significantly induced and peaked at 6 h under both 25 and 150 mM NaCl. Compared to the control, the expression of *PtSOS1* significantly increased by 5.8-folds, while that of *PtHKT1;5* increased only by 1.2-folds in roots under 25 mM NaCl; on the contrary, the expression of *PtSOS1* increased by 1.4-folds, whereas that of *PtHKT1;5* increased by 2.2-folds in roots under 150 mM NaCl. In addition, *PtNHX1* was induced instantaneously under 25 mM NaCl, while its expression was much higher and more persistent in shoots under 150 mM NaCl. These results provide stronger evidences for the previous hypothesis and extend the model which highlights that SOS1, HKT1;5, and NHX1 synergistically regulate Na^+^ homeostasis by controlling Na^+^ transport systems at the whole-plant level under both lower and higher salt conditions. Under mild salinity, PtNHX1 in shoots compartmentalized Na^+^ into vacuole slowly, and vacuole potential capacity for sequestering Na^+^ would enhance Na^+^ loading into the xylem of roots by PtSOS1 through feedback regulation; and consequently, Na^+^ could be transported from roots to shoots by transpiration stream for osmotic adjustment. While under severe salinity, Na^+^ was rapidly sequestrated into vacuoles of mesophyll cells by PtNHX1 and the vacuole capacity became saturated for sequestering more Na^+^, which in turn regulated long-distance Na^+^ transport from roots to shoots. As a result, the expression of *PtHKT1;5* was strongly induced so that the excessive Na^+^ was unloaded from xylem into xylem parenchyma cells by PtHKT1;5.

## Introduction

Soil salinity is one of the major environmental factors restricting agricultural productivity worldwide (Venema et al., 2002; Zhang et al., 2010; Flowers et al., 2015; Gu et al., 2016). According to Food and Agriculture Organization’s (FAO’s) Land and Plant Nutrition Management Service, over 800 million hectares of land are salt-affected (Munns and Tester, 2008), accounting for over 6% of the world land area (Arabbeigi et al., 2014). One of the major consequences of salt stress is a disruption of Na^+^ and K^+^ homeostasis in both cellular and whole-plant levels, accompanied by membrane dysfunction and attenuation of cellular metabolism, resulting in the inhibition of cell division, growth, photosynthesis, and development (Omielan et al., 1991; Flowers, 1999; Horie and Schroeder, 2004; Yan et al., 2013; Duan et al., 2015). To avoid Na^+^ toxicity, plants have evolved various adaptation mechanisms, such as restricting Na^+^ uptake from environments, extruding cytoplasmic Na^+^ to the outside of the cell and sequestering Na^+^ into vacuoles to reduce Na^+^ accumulation in cytosol (Wang et al., 2007; Hamed et al., 2013).

*Puccinellia tenuiflora* is the only halophytic species in Gramineae with excellent tolerance to salinity and mainly districted in the saline-alkali soil in north China and seaside (Wang et al., 2002, 2005). Our previous study showed that *P. tenuiflora* could maintain significantly lower net Na^+^ uptake rates than wheat, especially under 150 and 200 mM NaCl; the accumulation of Na^+^ in *P. tenuiflora* was increased, but was significantly lower than that in wheat under 50–200 mM NaCl. Meanwhile, *P. tenuiflora* maintained significantly higher tissue K^+^ concentrations under various concentrations of NaCl, indicating that restricting unidirectional Na^+^ influx in roots and maintaining a high selectivity for K^+^ over Na^+^ is a major salt-tolerance mechanism of *P. tenuiflora* (Wang et al., 2009). Therefore, extruding Na^+^ might be a crucial strategy for *P. tenuiflora* to overcome salinity, and its salt-tolerant phenotype might be facilitated by the interaction of several Na^+^ transport-relevant proteins, such as plasma membrane Na^+^/H^+^ antiporter SOS1, Na^+^ transporter HKT1;5, tonoplast Na^+^/H^+^ antiporter NHX1, etc. SOS1 functions mainly in loading Na^+^ from xylem parenchyma cells (XPCs) into xylem in roots and plays an important role in maintaining Na^+^ homeostasis in whole plant (Shi et al., 2002b; Qi and Spalding, 2004). It was suggested that *Arabidopsis thaliana* AtSOS1 controlled long-distance Na^+^ transport (Shi et al., 2002b). Similar result was obtained from tomato (*Solanum lycopersicum*) revealed that SlSOS1 was critical for the partitioning of Na^+^ among plant organs (Olías et al., 2009). Many researches demonstrated that some members of HKT transporters mediate Na^+^ transport and are involved in regulating Na^+^ and K^+^ homeostasis (Mäser et al., 2002b; Platten et al., 2006). In *Oryza sativa*, a typical Na^+^ selective transporter SKC1 (OsHKT1;5) plays a vital role in maintaining higher K^+^/Na^+^ ratio and improving salt tolerance (Ren et al., 2005). In *A. thaliana*, AtHKT1;1 unloads Na^+^ from xylem vessels to XPCs, thereby reducing Na^+^ content in leaves, and the overexpression of *AtHKT1;1* specifically in mature root stele increased the influx of Na^+^ into XPCs, leading to increased shoots Na^+^ exclusion (Mäser et al., 2002a,b; Sunarpi et al., 2005; Møller et al., 2009). In *Triticum monococcum*, TmHKT1;5-A localized on the plasma membrane of root cells surrounding xylem vessels contributes to withdrawing Na^+^ from the xylem and reducing transport of Na^+^ into leaves (Munns et al., 2012). It was suggested NHX1 sequesters Na^+^ into vacuoles and plays a major role in regulating cellular pH and Na^+^ homeostasis (Apse et al., 1999; Blumwald, 2000; Shi and Zhu, 2002). It has been demonstrated that the overexpression of *NHX1* improved salt tolerance in different plant species. For example, overexpression of *AtNHX1* in *A. thaliana* and *AgNHX1* from a halophyte *Atriplex gmelini* in *O. sativa* significantly increased the salt tolerance of the transgenic plants (Apse et al., 1999; Hamada et al., 2001; Ohta et al., 2002). Similarly, overexpressing *DmNHX1* from *Dendranthema morifolium* resulted in enhanced salt tolerance of transgenic *A. thaliana* (Zhang et al., 2012). Interestingly, in the salt-accumulating xero-halophyte *Zygophyllum xanthoxylum*, the transport ability of SOS1 exceeded that of HKT1;5 under 50 mM NaCl, Na^+^ was loaded into the xylem and transport to leaves, and then ZxNHX efficiently compartmentalized Na^+^ into vacuoles of leaves. However, the silencing of *ZxNHX* converted *Z. xanthoxylum* from a typical salt-accumulating plant to a salt-excluding plant; the transport ability of HKT1;5 exceeded that of SOS1 in *ZxNHX*-silenced line and the excessive Na^+^ was unloaded from xylem into XPCs; therefore, Na^+^ accumulation in shoots was restricted (Yuan et al., 2015). In consequence, SOS1, HKT1;5, and especially NHX1 together play essential roles in maintaining the salt-accumulation characteristic of halophytes (Yuan et al., 2015).

Based on our previous work, we proposed a hypothetical model that SOS1 functions in regulating Na^+^ transport system in the membrane of XPCs by loading Na^+^ under mild salt stress, while HKT is involved in Na^+^ retrieval from the xylem when plants are exposed to severe salt stress, according to the accumulation of Na^+^ and K^+^, and the salt stress-responsive expression of *PtSOS1* in *P. tenuiflora* (Guo et al., 2012). In the present study, we analyzed the expression patterns of *PtSOS1*, *PtHKT1;5*, and *PtNHX1* in *P. tenuiflora* under 25 and 150 mM NaCl and combined our previous work about its physiological characteristics under various NaCl treatments (Wang et al., 2009; Guo et al., 2012) to further confirm our previous hypothesis and extend the model.

## Materials and Methods

### Plant Growth Conditions and Treatments

The seeds of *P. tenuiflora* were grown on the hydroponic culture sieves covered with bibulous paper and wetted with distilled water in rectangular trays in dark for 7 days. After germination, seedlings were irrigated with Hoagland nutrient solution (5 mM L^-1^ KNO_3_, 0.5 mM L^-1^ NH_4_H_2_PO_4_, 0.25 mM L^-1^ MgSO_4_.7H_2_O, 1.5 mM L^-1^ Ca(NO_3_)_2_.4H_2_O, 0.5 mM L^-1^ Fe-citrate, 92 μM L^-1^ H_3_BO_3_, 18 μM L^-1^ MnCl_2_.4H_2_O, 1.6 μM L^-1^ ZnSO_4_.7H_2_O, 0.6 μM L^-1^ CuSO_4_.5H_2_O, 0.7 μM L^-1^ (NH_4_)_6_Mo_7_O_24_.4H_2_O) that was renewed every 3 days. When seedling reached 5 cm in height, they were transferred into black containers with the Hoagland nutrient solution and cultivated in a greenhouse with the temperature of 28°C/23°C (day/night), the daily photoperiod of 16/8 h (light/dark, the flux density of about 600 μmol m^-2^ s^-1^) and the relative humidity of 60–80%. Solution was renewed every 3 days. Given that the growth of *P. tenuiflora* was inhibited slightly when external NaCl concentration increased to 25 mM, and became severely under 150 mM NaCl treatments (Wang et al., 2009, 2015; Guo et al., 2012), 4-week-old seedlings were treated with 25 (mild salt stress) or 150 mM NaCl (severe salt stress) for 0, 1, 6, 24, and 48 h before harvest.

### RNA Extraction and cDNA Synthesis

Total RNA was extracted from roots and shoots of above harvested seedlings using the RNAprep pure plant Kit (Tiangen Biotech Co., Ltd, Beijing, China). The first-strand cDNA was synthesized with PrimeScript^TM^ RT Master Mix (Perfect Real Time; Takara Biotech Co., Ltd, Dalian, China).

### Real-time Quantitative Polymerase Chain Reaction (qRT-PCR) Analysis

The primers of *PtACTIN*, *PtSOS1*, *PtHKT1;5*, and *PtNHX1* were designed by using the Primer 5.0 program (Premier Biosoft International, Palo Alto, CA, USA; **Table 1**). The expression levels of *SOS1*, *HKT1;5*, and *NHX1* genes in different tissues of *P. tenuiflora* under different salt treatments (25 or 150 mM NaCl) were detected by ABI PRISM 7500 sequence detection system. *PtACTIN* was used for RNA normalization. SYBR Green PCR master mix (Takara Biotech Co., Ltd, Dalian, China) was used for 20 μL PCR with the program as follows: 95°C for 30 s, 40 cycles of 95°C for 5 s, and 60°C for 34 s. All reactions were performed with three replicates. The relative expression levels of all genes were calculated using the 2^-ΔΔCt^ method (Zhang et al., 2016).

**Table 1:** Sequences of primers used for quantitative real-time PCR amplification.

Primer	Sequence (5′–3′)	Gene
P1	TTGACTACGACCAGGAGATGGA	*PtACTIN*-F
P2	TGAAGGATGGCTGGAAGAGG	*PtACTIN*-R
P3	GACGAATAACTCAATCCACAGCAA	*PtSOS1*-F
P4	ACCGCAAACCCTTCCAATC	*PtSOS1*-R
P5	GGACCTCTCCACCTTGTCGT	*PtHKT1;5*-F
P6	CTGCTACCGTTTGTTTGTCACTCT	*PtHKT1;5*-R
P7	GCAATGAACTCCGCAATGATAC	*PtNHX1*-F
P8	GCTGTAATGCTTCCTTCTCTTCCT	*PtNHX1*-R


### Statistical Analysis

All the data were presented as means with standard deviation, and data analysis was performed by one-way analysis of variance (ANOVA) using SPSS Statistics 19 software (SPSS Inc., Chicago, IL, USA), Duncan’s multiple range test was used to detect the differences among means at a significance level of *P* < 0.05.

## Results

### The Tissue-specific Expressions of *PtHKT1;5* and *PtNHX1*

**FIGURE 1:**
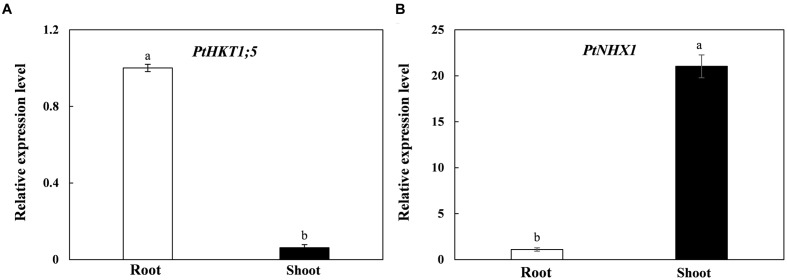
**The relative expression levels of *PtHKT1;5*** **(A)** and *PtNHX1* **(B)** in roots and shoots of *P. tenuiflora* under control condition (no additional NaCl). *ACTIN* was used as an internal reference. Experiments were repeated at least three times. Values are means ± standard deviations (SDs) (*n* = 3) and bars indicate SDs. Columns with different letters indicate a significant difference at *P* < 0.05 (Duncan’s test).

In order to investigate the tissue-specific expressions of *PtHKT1;5* and *PtNHX1* in *P. tenuiflora*, we performed qRT-PCR to test the relative expressions of the two genes in shoots and roots. The results showed that *PtHKT1;5* expressed mainly in roots and was barely detected in shoots (**Figure 1A**); however, the expression level of *PtNHX1* in shoots was significantly higher than that in roots (**Figure 1B**).

### The Expression Patterns of *PtSOS1* and *PtHKT1;5* in Roots under Different Concentrations of NaCl

**FIGURE 2:**
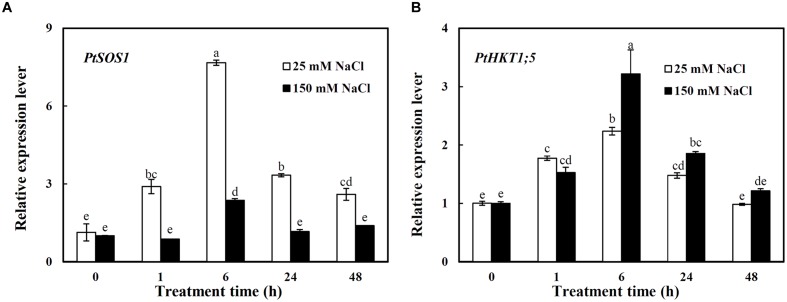
**The relative expression levels of *PtSOS1*** **(A)** and *PtHKT1;5* **(B)** in roots of *P. tenuiflora* under different concentration NaCl (25 and 150 Mm) for 0, 1, 6, 24, and 48 h. *ACTIN* was used as an internal reference. Experiments were repeated at least three times. Values are means ± SDs (*n* = 3) and bars indicate SDs. Columns with different letters indicate significant differences at *P* < 0.05 (Duncan’s test).

The expressions of *PtSOS1* and *PtHKT1;5* in roots were investigated under different concentrations of NaCl (**Figure 2**). Under 25 mM NaCl, the expression of *PtSOS1* showed a sharp increase and reached the peak value at 6 h which was 5.8-folds higher than that under control condition (0 h) and then a decrease trend, still 1.3-folds higher than that of control at 48 h (**Figure 2A**). The 150 mM NaCl significantly induced the expression of *PtSOS1* only by 1.4-folds at 6 h compared to control, then it decreased to the same expression level as control at 24–48 h (**Figure 2A**). Notably, the expression level of *PtSOS1* was always significantly higher under 25 mM NaCl than that under 150 mM NaCl from 1 to 48 h of treatments (**Figure 2A**).

*PtHKT1;5* was induced by both 25 and 150 mM NaCl with a similar pattern: the transcription levels of *PtHKT1;5* showed an increase before 6 h then a decrease trend, dropping to the control level at 48 h (**Figure 2B**). It is worth noting that the expression of *PtHKT1;5* was induced by 2.2-folds under 150 mM NaCl while by 1.2-folds under 25 mM NaCl at 6 h compared to control (**Figure 2B**).

### The Expression Pattern of *PtNHX1* in Shoots under Different Concentrations of NaCl

**FIGURE 3:**
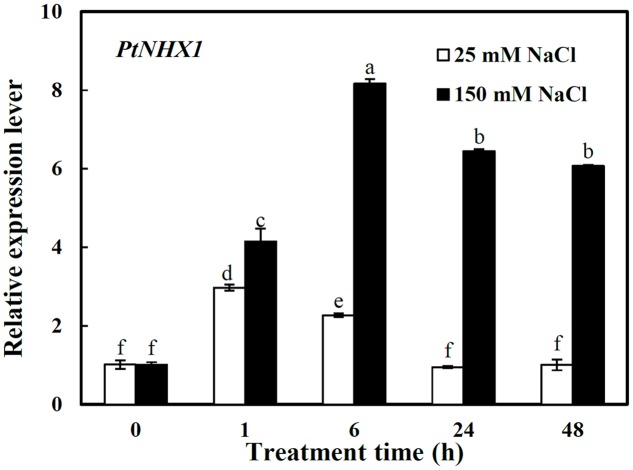
**The relative expression levels of *PtNHX1* in shoots of *P. tenuiflora* under different concentration NaCl (25 and 150 Mm) for 0, 1, 6, 24, and 48 h.** *ACTIN* was used as an internal reference. Experiments were repeated at least three times. Values are means ± SDs (*n* = 3) and bars indicate SDs. Columns with different letters indicate significant differences at *P* < 0.05 (Duncan’s test).

Here the relative expression level of *PtNHX1* was analyzed only in shoots since above result showed that it expressed dominantly in shoots (**Figure 1B**). *PtNHX1* in shoots were up-regulated by 25 and 150 mM NaCl with apparent different patterns (**Figure 3**). Under 25 mM NaCl, the transcript of *PtNHX1* was significantly increased, peaking at 1 h and 1.9-folds higher than control, then dropped to control level at 24 h after treatment (**Figure 3**). Under 150 mM NaCl, the transcript of *PtNHX1* was rapidly up-regulated and reached a maximum level at 6 h, 7.1-folds higher than control, and maintained significantly 5.4- and 5-folds higher levels at 24 and 48 h after treatment, respectively (**Figure 3**). In general, the transcript of *PtNHX1* under 150 mM NaCl was much higher than that under 25 mM NaCl from 1 to 48 h of treatments, especially, 2.6-, 5.8-, and 5.1-folds higher at 6, 24, and 48 h after treatments, respectively (**Figure 3**).

## Discussion

### PtSOS1 Plays a Vital Role in Loading Na^+^ Into Xylem in Roots Mainly under Mild Salt Condition

Plasma membrane Na^+^/H^+^ transporter SOS1 play important roles in Na^+^ transport. It has been found that *AtSOS1* was expressed mainly in roots and significantly induced by NaCl (Shi et al., 2000, 2002b). Similar expression pattern was observed for *O. sativa OsSOS1* (Martínez-Atienza et al., 2007), *Triticum aestivum TaSOS1* (Xu et al., 2008), *Phragmites australis PhaNHA1* (Takahashi et al., 2009), *Thellungiella salsuginea ThSOS1* (Oh et al., 2009), etc. Our previous studies showed that *PtSOS1* exhibited a higher expression level in roots (Guo et al., 2012), and the present study also showed that its expression in roots was strongly up-regulated by NaCl (**Figure 2A**). Shi et al. (2002b) found that *AtSOS1* preferentially expressed in parenchyma cells at the xylem/symplast boundary of roots, the encoding protein functions in loading Na^+^ into xylem for controlling Na^+^ delivery to the shoots and storage in leaf mesophyll cells in *A. thaliana*, and the *atsos1* mutant accumulated less Na^+^ in shoots than the Wild Type (WT) under moderate salinity. Similar result was obtained from the xerophyte *Z. xanthoxylum*: *ZxSOS1*-silenced plants accumulated more Na^+^ in roots but less in shoots than WT under 50 mM NaCl, indicating that ZxSOS1 was involved in long-distance transport and spatial distribution of Na^+^ among plant tissues (Ma et al., 2014). In the present study, the expression of *PtSOS1* displayed a rapid and persistent increase trend under mild salinity, but an instantaneously (only at 6 h) increase under severe salt treatment (**Figure 2A**), suggesting that PtSOS1 functions in delivery Na^+^ to shoots for osmotic adjustment by loading Na^+^ into the xylem mainly under 25 mM NaCl (Shi et al., 2002b; Guo et al., 2012; Yuan et al., 2015). While under 150 mM NaCl, excessive Na^+^ was accumulated in shoots, which in turn restricted long-distance Na^+^ transport from roots to shoots by reducing Na^+^ loading into the xylem (Guo et al., 2012).

### PtHKT1;5 Mediate Na^+^ Unloading from Xylem Vessels in Roots Dominantly under Severe Salt Stress

HKT-like proteins are known to play significant roles in regulating Na^+^ and K^+^ transport and maintaining their homeostasis in higher plants (Schachtman and Schroeder, 1994; Munns and Tester, 2008; Horie et al., 2009). HKT transporters encoded by *HKT1;5*-like gene were identified as Na^+^ transporters and mediate Na^+^ retrieval from xylem in rice (Ren et al., 2005) and wheat (Byrt et al., 2007). It is noteworthy that *OsHKT1;5* is preferentially expressed in the parenchyma cells surrounding the xylem vessels of roots and the voltage-clamp analysis showed that OsHKT1;5 mediates Na^+^ exclusion from leaves by removing Na^+^ from the xylem sap in roots to prevent Na^+^ over-accumulation in shoots (Ren et al., 2005). Moreover, Sunarpi et al. (2005) reported that AtHKT1, renamed as AtHKT1;1, selectively unloaded Na^+^ directly from xylem vessels to XPCs and thus reduced Na^+^ content in xylem vessels in roots and leaves, thereby playing a crucial role in protecting leaves from Na^+^ toxicity. Similar results were observed for Nax2 (TmHKT1;5-A) and Kna1 (TaHKT1;5-D) involved in limiting massive Na^+^ transport from xylem to leaves (Munns et al., 2012). *TmHKT1;5-A* and *TaHKT1;5-D* were both expressed in the roots but not in the leaves of *T. monococcum* and *T. aestivum*, respectively, and the expression of *TmHKT1;5-A* was up-regulated by salinity (Byrt et al., 2007; Munns et al., 2012). Our present results also showed that *PtHKT1;5* mainly expressed in roots (**Figure 1A**), and it was up-regulated by NaCl, especially at higher concentration (150 mM) (**Figure 2B**), implying PtHKT1;5 plays an important role in unloading Na^+^ from xylem to parenchyma cells in roots mainly under high concentration of NaCl. Guo et al. (2012) found that when *P. tenuiflora* was exposed to severe salt conditions, Na^+^ in vacuoles of its leaves reached the maximum concentration, which in turn regulated HKT to unload Na^+^ from xylem. Our results further confirmed this opinion: shoot Na^+^ accumulation in *P. tenuiflora* was significantly increased in *P. tenuiflora* under 150 mM NaCl (Wang et al., 2009), which in turn strongly induced the expression of *PtHKT1;5*, facilitated excessive Na^+^ unloading into XPCs of roots (**Figure 2B**), and consequently alleviated Na^+^ toxicity in plants.

### PtNHX1 Plays a Crucial Role in Compartmentation of Na^+^ into Vacuoles in Shoots under Mild and Severe Salinity

Sequestering Na^+^ into vacuoles is one of crucial strategies for plants to reduce Na^+^ toxicity in cytoplasm under salt stress (Nass et al., 1997; Martinoia et al., 2000; Gong et al., 2005). The tonoplast Na^+^/H^+^ antiporter NHX1 is a ubiquitous transmembrane protein playing a key role in compartmentalizing Na^+^ into vacuoles to maintain Na^+^ homeostasis and thus to enhance plant salt tolerance (Shi and Zhu, 2002). Many studies revealed that the transcript level of *NHX1* in shoots was observably increased during salt treatments. The transcript level of *Mesembryanthemum crystallinum McNHX1* increased and reached a high and stable level in leaves but not in roots 10 days after onset of salt stress (Cosentino et al., 2010). A similar trend was observed in cotton and chrysanthemum (Wu et al., 2004; Zhang et al., 2012). Wu et al. (2011) found that *ZxNHX* also preferentially expressed in the leaf tissue and was significantly induced by salt treatments. In *Z. xanthoxylum* and *D. morifolium*, a positive correlation existed between the expression of *NHX1* and Na^+^ accumulation in leaves when plants were exposed to salinity (Wu et al., 2011; Zhang et al., 2012). In this study, although the expression of *PtNHX1* in shoots was significantly up-regulated by both 25 and 150 mM NaCl (peaked at 1 and 6 h after treatments, 1.9- and 7.1-folds higher than that of control), its expression under 150 mM NaCl was 2.6-, 5.8-, and 5.1-folds higher than that under 25 mM NaCl at 6, 24, and 48 h, respectively (**Figure 3**), indicating that PtNHX1 sequestrated Na^+^ in shoots mainly under higher NaCl concentration. It was proposed that under mild salinity, Na^+^ accumulation in the leaves of plants was probably below vacuole capacity for sequestering Na^+^ (Blumwald et al., 2000), therefore, in our study *PtNHX1* was induced transiently (**Figure 3**). Under severe salinity, excessive Na^+^ was accumulated in shoots (Wang et al., 2009), here, *PtNHX1* expressed higher and more persistently (**Figure 3**) so that a great quantity of Na^+^ could be sequestered into the vacuoles as soon as possible.

### The Model of Na^+^-transporter Regulating Na^+^ Homeostasis at Whole-plant Level under Different Salt Concentrations

The survival of plants under salinity was attributed to many genes and a complex genetic regulatory network, including extruding excessive Na^+^ or sequestering Na^+^ into vacuoles and controlling Na^+^ long-distance transport (Shi and Zhu, 2002; Shi et al., 2002a,b; Ren et al., 2005; Apse and Blumwald, 2007; Ghars et al., 2008). These processes were controlled by corresponding Na^+^ transporters. It was confirmed that SOS1 and HKT1;5 have opposite roles in regulating Na^+^ transport from roots to shoots by mediating Na^+^ efflux and influx, respectively, across the plasma membranes of XPCs, hence, the cooperation of them contributes to Na^+^ homeostasis in the whole plant (Hauser and Horie, 2010; Guo et al., 2012; Yuan et al., 2015). Moreover, it was found that *Z. xanthoxylum* ZxNHX determined Na^+^ accumulation in vacuoles of mesophyll cells and controlled the opposite Na^+^ fluxes across the plasma membranes of XPCs mediated by ZxSOS1 and ZxHKT1;1 through a feedback regulation (Yuan et al., 2015). Our previous study showed that under 25 mM NaCl, *P. tenuiflora* maintained a significantly lower net Na^+^ uptake rate and there was no significant increase in Na^+^ accumulation (Wang et al., 2009). Under the same condition, here, *PtNHX1* was induced transiently (**Figure 3**), indicating that Na^+^ was compartmentalized into vacuoles slowly. While under severe salt condition (150 mM NaCl), the net Na^+^ uptake rate and Na^+^ concentration increased dramatically in tissues of *P. tenuiflora* (Wang et al., 2009). Here, *PtNHX1* expressed higher and more persistently (**Figure 3**), indicating that a great quantity of Na^+^ could be sequestered into vacuoles rapidly. The expressions of *PtSOS1* and *PtHKT1;5* in roots were significantly induced and peaked at 6 h after both 25 and 150 mM NaCl treatments, but the expression of *PtSOS1* was 5.8-folds whereas that of *PtHKT1;5* 1.2-folds higher under 25 mM NaCl than under control condition (**Figure 2**). It was proposed that under mild salinity, Na^+^ accumulation in leaves is below vacuole capacity for sequestering Na^+^ by NHX1 (Blumwald et al., 2000; Wang et al., 2009), thus, Na^+^ transport ability of SOS1 outweigh that of HKT1;5 and Na^+^ could be loaded into the xylem (Guo et al., 2012; Yuan et al., 2015). On the contrary, the transcript of *PtSOS1* increased by only 1.4-folds whereas that of *PtHKT1;5* by 2.2-folds in roots 6 h after plants were treated with 150 mM NaCl (**Figure 2**), suggesting that under severe salinity, Na^+^ accumulation reached or even exceeded vacuole capacity for sequestering Na^+^ in shoots and the transport ability of HKT1;5 overwhelm SOS1 so that Na^+^ could be unloaded from xylem into XPCs (Guo et al., 2012; Yuan et al., 2015).

## Conclusion

**FIGURE 4:**
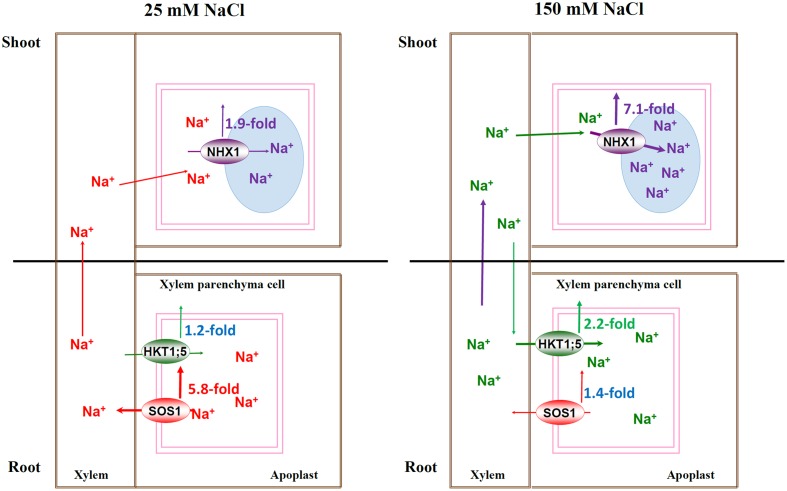
**The schematic model for Na^+^-transporters regulating Na^+^ homeostasis under mild and severe salinity at whole-plant level.** Under mild salinity, PtNHX1 in shoots compartmentalized Na^+^ into vacuoles slowly, and vacuole potential capacity for sequestering Na^+^ would enhance Na^+^ loading into the xylem by PtSOS1, then, Na^+^ could be transported to shoots by transpiration stream; under severe salinity, Na^+^ was rapidly and persistently sequestrated in vacuoles of leaves by PtNHX1 and the vacuole capacity became saturated for sequestering Na^+^ rapidly, which in turn restricted Na^+^ long-distance transport from roots and strongly induced the expression level of *PtHKT1;5* to facilitate unloading excessive Na^+^ from xylem into XPCs, and consequently, to alleviate Na^+^ toxicity in photosynthetic tissues.

Our results provided a stronger evidence for the previous hypothesis and further extended the model which highlights that SOS1, HKT1;5, and NHX1 synergistically regulate Na^+^ homeostasis by controlling Na^+^ transport systems at the whole-plant level under both lower and higher salt conditions. Under mild salinity, PtNHX1 in shoots compartmentalizes Na^+^ into vacuole slowly, and leaf vacuole potential capacity for sequestering Na^+^ will enhance Na^+^ loading into the xylem of roots by PtSOS1 through feedback regulation, and consequently, Na^+^ could be transported into shoots by transpiration stream for osmotic adjustment. However, under severe salinity, Na^+^ was rapidly and persistently sequestrated into vacuoles of mesophyll cells by PtNHX1 and leaf vacuole capacity become saturated very soon for sequestering Na^+^, which in turn restricts long-distance Na^+^ transport from roots to shoots, induce the expression of *PtHKT1;5* to facilitate excessive Na^+^ unloading from xylem into XPCs, and consequently, and alleviates Na^+^ toxicity in photosynthetic tissues (**Figure 4**). In summary, PtSOS1, PtHKT1;5, and PtNHX1 play very important roles in synergistically regulating Na^+^ homeostasis by controlling Na^+^ transport systems at the whole-plant level in the halophytic grass *P. tenuiflora* under salt conditions, therefore, this model exists an extensive of application prospect. In addition, as a halophytic grass and the only halophyte plant in Gramineae, *P. tenuiflora* shares the similar salt-exclusion mechanism to cereals with the close genetic relationship, while the former has much stronger salt exclusion ability. Thus, these genes from *P. tenuiflora* could be transformed into cereal crops for potential salt tolerance improvement.

## Author Contributions

S-MW and QM designed the research; L-JD and W-DZ performed the experiments; W-DZ, PW, ZB, S-MW, QM, J-LZ, and A-KB analyzed data and wrote the manuscript. All the authors agreed on the contents of the paper and posted no conflicting interest.

## Conflict of Interest Statement

The authors declare that the research was conducted in the absence of any commercial or financial relationships that could be construed as a potential conflict of interest.
